# High-throughput characterization of protein–RNA interactions

**DOI:** 10.1093/bfgp/elu047

**Published:** 2014-12-13

**Authors:** Kate B. Cook, Timothy R. Hughes, Quaid D. Morris

**Keywords:** RNA-binding proteins, RBP target identification, high-throughput sequencing, RNA secondary structure

## Abstract

RNA-binding proteins (RBPs) are important regulators of eukaryotic gene expression. Genomes typically encode dozens to hundreds of proteins containing RNA-binding domains, which collectively recognize diverse RNA sequences and structures. Recent advances in high-throughput methods for assaying the targets of RBPs *in vitro* and *in vivo* allow large-scale derivation of RNA-binding motifs as well as determination of RNA–protein interactions in living cells. In parallel, many computational methods have been developed to analyze and interpret these data. The interplay between RNA secondary structure and RBP binding has also been a growing theme. Integrating RNA–protein interaction data with observations of post-transcriptional regulation will enhance our understanding of the roles of these important proteins.

## INTRODUCTION: RNA-BINDING PROTEINS, RNA-BINDING DOMAINS AND RNA SECONDARY STRUCTURE

RNA-binding proteins (RBPs) have diverse roles in post-transcriptional gene expression, including regulation of alternative splicing, RNA export and localization, RNA stability and translation [1]. Many RBPs regulate multiple cellular processes [2–5], and are implicated in human diseases including cancer and neurological disorders [6]. RBP functionality in gene regulation is naturally dependent on their ability to selectively recognize and bind target RNAs within the cell; consequently, elucidation of RBP specificity is an area of active research. Recent technological developments have allowed characterization of RNA–protein interactions at an unprecedented scale.

Here we introduce the major classes of sequence- and structure-specific RNPs, and discuss aspects of RNA secondary structure that impact RBP binding; knowledge of how proteins interact with RNA is important for the interpretation of high-throughput data. We then review current methods for high-throughput experimental determination of the RNA targets of RBPs *in vitro* and *in vivo*, as well as methods to determine the proteins bound to an RNA molecule. Finally, we discuss computational methods for analyzing high-throughput data and predicting RBP binding.

### RNA recognition by RNA-binding domains

**Figure 1:**
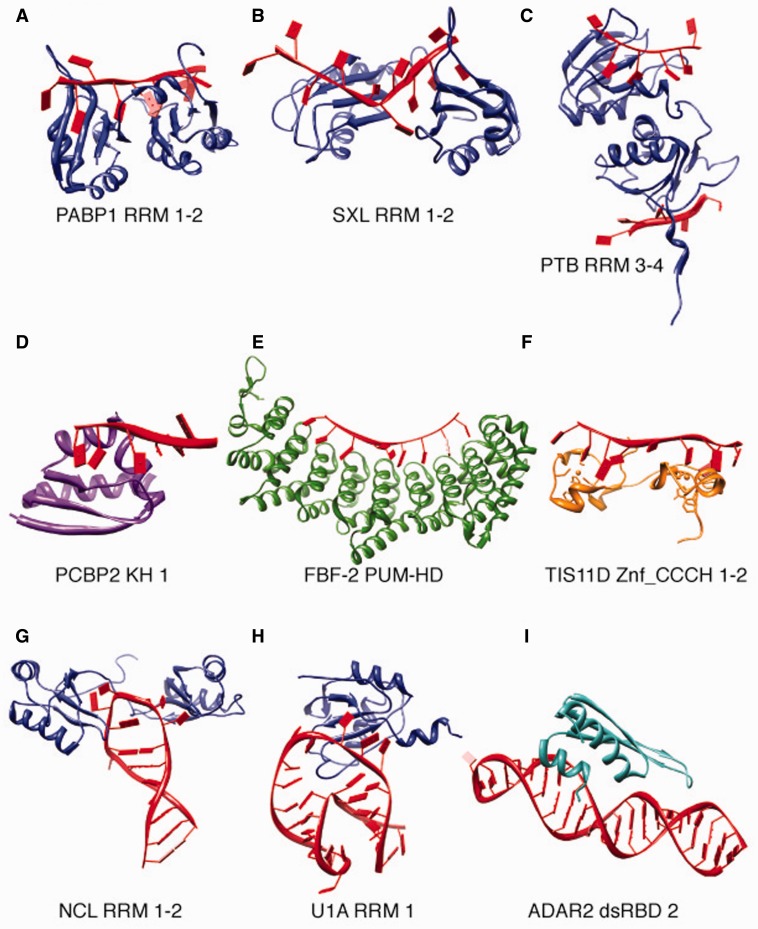
RNA-binding domains use a variety of strategies for binding RNA. (**A–C**), Different arrangements of two RRM domains. (A) RRMs 1–2 of PABP1 are arranged to form a flat RNA-binding surface (PDB ID: ICVJ). (B) RRMs 1–2 of SXL form an RNA-binding cleft (1B7F). (C) RRMs 3–4 of PTB are arranged back to back (2ADC). (**D–F**) Examples of other RNA-binding domains. (D) KH domain 1 of PCBP2 forms an RNA-binding cleft (2PY9). (E) The Puf repeats of the FBF-2 PUM-HD form a concave RNA-binding surface (3K62). (F) The two CCCH zinc fingers of TIS11D/ZFP36L2 (1RGO). (**G–I)** RBPs binding to structured RNA. (G) Hairpin loop recognition by RRMs 1–2 of Nucleolin (1RKJ). (H) Bulge loop recognition by RRM 1 of U1A/SNRPA (1AUD). (I) dsRNA binding by the dsRBD domain of ADAR2 (2L2K).

Different classes of RNA-binding domains (RBDs) use different strategies for binding to RNA. Nonetheless, there are some general features of RBP–RNA interactions: RNA recognition is commonly a combination of recognition of the RNA by the overall protein fold (involving hydrogen bonds with backbone atoms) as well as specific amino acid side chain–nucleotide interactions. Target specificity is often accomplished by way of hydrogen bonding and electrostatic interactions; the latter also contribute to the protein’s affinity for RNA along with stacking interactions [7]. Individual RNA-binding domains typically only contact a few nucleotides, and combinations of RBDs within the same protein are frequently observed, presumably to increase affinity and specificity. Structures of the most common RNA-binding domains are shown in Figure 1 and described below. Not all sequence-specific RBPs contain canonical RBDs [8, 9]. The full extent of the RNA-binding proteome is an area of current research, discussed in a further section.

#### RNA recognition motif

The RNA recognition motif (RRM) is found in all domains of life; in metazoans, it is the most common RNA-binding domain. A single RRM spans ∼90 amino acids and contains two conserved motifs, RNP-1 and RNP-2, consisting respectively of 8 and 6 mostly positively charged or aromatic amino acids [10, 11].

Structurally, the RRM consists of a four-strand antiparallel β-sheet backed by two α-helices. RRMs are highly versatile in their mode of RNA recognition: in canonical RRM–RNA interactions, the bound RNA lies across the β-sheet and contacts one or more key residues in the conserved RNP-1 and RNP-2 motifs; however, the amount of the β-sheet surface directly contacting the RNA varies considerably [12]. Noncanonical RRM–RNA interactions can involve interactions with loop regions or amino acids N- or C-terminal to the RRM domain. In some cases, the β-sheet surface is not involved in RNA binding at all [13].

The β-sheet surface of a single RRM can contact up to four nucleotides, while engaging the loop regions external to the β-sheet can allow binding of up to six nucleotides [12]. Approximately 40% of RRM-containing proteins contain multiple RRM domains [12, 14]. Two RRMs can be separated by a flexible linker, can be arranged as a continuous RNA-binding platform either oriented in the same direction (Figure 1A, [15]) or forming an RNA-binding cleft (Figure 1B, [16]) or can interact back to back, forcing the RNA to loop around the protein (Figure 1C, [17]). Presumably, other binding modes are possible; as in Nucleolin, U1A/SNRPA [18] or other proteins that bind RNA in the context of secondary structures (Figure 1G–H).

#### K homology domain

K homology (KH) domains, named after the founding member of the family, hnRNP K [19], are also widely present in all three domains of life, although the KH type most commonly found in prokaryotes adopts a different fold than the majority eukaryotic type [20]. The typical metazoan genome encodes several dozen proteins containing KH domains [14]. KH domains are about 70 amino acids in size, and bind RNA inside a cleft composed of two α-helices, a variable loop sequence containing a conserved GXXG motif, and a β-strand (Figure 1D). This binding cleft can accommodate four RNA bases, and KH domains are often combined in multiples to enhance affinity and specificity of binding [21].

#### Double-stranded RNA binding domain

Double-stranded RNA binding domains (dsRBDs) are involved in diverse aspects of post-transcriptional regulation, including RNA editing [22], miRNA biogenesis [23] and RNA localization [24, 25]. The human genome encodes 18 proteins containing dsRBDs. The domain is 65–70 amino acids in size and consists of two α-helices packed against a three-strand antiparallel β-sheet. Portions of both α-helices and a loop region between two of the β-strands are involved in RNA binding, and mutation of the amino acids in these regions can affect binding [24, 26]. Structural details of the dsRBD–RNA interaction were recently and extensively reviewed in [27].

Because of the nature of the A-form RNA double helix, in which the major groove is narrow and deep[28], it is generally assumed that dsRBDs recognize only the double-stranded RNA (dsRNA) shape and are not sequence specific. Nonetheless, dsRBD containing proteins do recognize specific target RNAs, which may be the result of recognizing mismatches or bulges in RNA duplexes: a recent study of Staufen targets observed that dsRNA stems with specific numbers of base pairs and few unbalanced unpaired bases were enriched in the bound transcripts [25]. Intriguingly, a structure of ADAR2 bound to RNA (Figure 1I) displays sequence-specific contacts between the protein and the minor groove [29].

#### Pumilio homology domain

In contrast to the other major RNA binding domains, for which the elucidation of a recognition code has proven elusive, RNA recognition by Pumilio Homology Domains (PUM-HD) is understood to the extent that custom proteins can be designed to bind new sequences. The PUM-HD typically consists of 8 PUF (Pumilio and FBF) repeats of a 36 amino acid motif, and the entire domain forms a curved structure that binds RNA in the concave side of the domain, while the convex side mediates protein–protein interactions [30]. Each PUF repeat contacts two RNA nucleotides, and recognizes its target nucleotides using only a few well-conserved amino acids. Because of its modular design, a recognition code for the PUF repeat has been developed, and custom PUM-HD domains have been designed to bind new motifs [31]. PUM-HD domains have also been engineered to bind to cytosine (which is not observed in any of the natural PUF binding specificities) and to bind targets longer than 8 bases by increasing the number of repeats [32, 33].

#### Zinc fingers

Zinc fingers are a large and diverse class of domains with the common property of coordinating zinc. The different types of zinc fingers have varying three-dimensional structure and likely have independent evolutionary origins. Nonetheless, several types act as DNA-, RNA-, and protein-binding domains. The extent to which individual zinc finger proteins bind and recognize each class of biopolymers is unknown, however there are some trends: C2H2 zinc fingers are usually DNA binding, while CCCH zinc fingers are primarily single-stranded RNA binding. CCHC zinc knuckles in viral and metazoan proteins also bind RNA; however, RNA binding by metazoan CCHC zinc knuckles is understood only in the context of proteins that also contain another RBD [14, 34]. In support of these trends, a recent mass spectrometry-based study (see below) observed significant enrichment for CCHC, CCCH and several smaller zinc finger families, but not C2H2 zinc fingers, in the mRNA-bound proteome [35].

The human genome encodes ∼60 proteins with CCCH zinc fingers (a larger number than contain KH domains), of which 11 have evidence of single-stranded RNA (ssRNA) binding [14]. RNA recognition by CCCH proteins is accomplished through stacking interactions and hydrogen bonds, particularly between the RNA and backbone atoms, so the overall fold of the protein is likely to be important for RNA recognition [36].

### The role of RNA secondary structure in RBP binding

RNA structure is a critical aspect of describing, measuring and predicting RBP binding: many RBPs recognize specific structures, while those that bind single-stranded RNA presumably compete with RNA structures. In addition, RBP binding likely has some impact on RNA structure, and *in vivo* RNA structure is impacted both by the presence of ATP-dependent RNA helicases, as well as a number of proteins that could bind co-transcriptionally. Despite the development of high-throughput methods for measurement of RNA structure, there is ongoing controversy regarding the degree of RNA structure present in cells and the accuracy of both computational algorithms and these experimental techniques to predict RNA folding.

#### Experimental determination of RNA secondary structure

The secondary structure of an RNA molecule can be determined by footprinting techniques: cutting the RNA using RNases specific to ssRNA or dsRNA, or small molecule reagents that cleave or modify RNA at positions in a manner proportional to their accessibility [37]. The cleaved or modified sequences are traditionally separated on a sequencing gel to determine the positions of more or less accessible nucleotides. The first genome-wide application of this strategy was FragSeq, which consists of fragmenting RNA using nuclease S1 (preferring ss or accessible RNA), ligating adaptors to the 5′ phosphate produced and high-throughput sequencing to identify cleavage locations [38]. A similar method, PARS, uses fragmentation with two complementary enzymes: RNase V1, which preferentially cleaves dsRNA, and nuclease S1. The PARS score is the log of the ratio of V1/S1 reads at each position, and reflects the tendency for that base to be double-stranded [39]. Small molecule reagents have also been used in a genome-wide fashion. Dimethyl sulphate (DMS) has been used to profile RNA secondary structure *in vivo* in *Arabidopsis *[40] and yeast and human cells [41].

The overall trends identified by these studies vary. RNA accessibility around the start codon was associated with translational efficiency (as measured by ribosome profiling) in yeast using PARS [39] and in *Arabidopsis* using DMS [40]; however, overall mRNA structural accessibility did not correlate with translation efficiency in yeast using DMS [41]. Using PARS, Kertesz et al. observed a higher level of base pairing in yeast coding sequences as compared with untranslated regions [39]. This contrasts with the results obtained for human PARS data [42] as well as data from *Arabidopsis* using DMS [40] all of which observe coding regions as being more single stranded. Computational predictions in both yeast (K.B.C., unpublished observation) and mammals [43] support a relatively less structured coding sequence on average. One possible application of these methods is to determine the impact of protein binding on RNA secondary structure, as binding by both ssRNA-preferring RBPs and RBPs that recognize structured RNA is likely to have an impact on RNA structure in an ‘induced fit’ fashion.

#### Computational prediction of RNA secondary structure

Computational prediction of mRNA secondary structure generally conforms to one of the two approaches. The first relies on the assumption that thermodynamically stable structures are more likely to exist than unstable structures, exemplified by the Zuker MFOLD algorithm [44] and extended using approaches that consider all possible structures using partition function approaches [45–47]. Many of these algorithms have been implemented in various packages including the Vienna RNA package [48] and the RNAstructure Web servers [49].

As an alternative to the free-energy based algorithms, covariation-based approaches take advantage of the fact that functional RNA secondary structures are more likely to be conserved through evolution. Covariation algorithms use a number of simplifying heuristics (reviewed in [50]), as simultaneous folding and alignment of RNA sequences is computationally costly [51]. While covariation algorithms have been successfully applied to define many noncoding RNA families [52], large numbers of related sequences are required for input. As well, care must be taken in interpreting the results, as the results from covariation methods may be affected by the choice of alignment method if it is not selected to minimize spurious alignments [53], and covariation methods may over-predict structure because their statistical scoring procedure is biased toward predicting base pairing [54, 55].

#### Benchmarking the accuracy of mRNA secondary structure estimates

Given the inconsistencies among the experimental methods for assessing mRNA secondary structure and uncertainty about the accuracy of computational predictions, it is important to evaluate the accuracy of both these types of estimates. However, doing so has been troublesome because of the lack of gold standards for mRNA secondary structures. Classic RNA secondary structure benchmarks are likely inappropriate because they are composed of highly structured ncRNAs like ribosomal RNAs and ribozymes. In addition, mRNAs are longer than most well-characterized ncRNAs, such that windowed approaches (e.g. the RNAplfold algorithm) are often preferred both for their speed and potentially increased accuracy [56].

Lange and colleagues performed an analysis to determine the accuracy of predicted secondary structures using yeast PARS data [39] and a curated set of structured cis-regulatory elements, and found that more accurate secondary structures were predicted using local (i.e. windowed) folding with window sizes of 100-150 nt, and that the edges of windows were predicted with less accuracy [57]. Estimates for the optimal window size based on siRNA efficacy (which depends on the accessibility of the target RNA) range from 80 nt to 800 nt [58, 59]. Li, Kazan and colleagues applied the observation that accessible sites are more likely to be bound by RBPs to compare the ability of PARS and the RNAplfold algorithm to score accessible sequences and separate bound and unbound transcripts from RIP-chip data [60]. RNAplfold performed significantly better except in the case when only nucleotides with robust PARS data were considered, at which point the difference was not statistically significant. A method that combined PARS data and computational predictions [61] performed better than RNAplfold on some RBPs but results on the entire benchmark were not reported. As such, because of the shallowness of current high-throughput experimental techniques for RNA secondary structure determination and the indirect nature of the data produced, it is likely that computational predictions will continue to be important. However, the optimal parameters (i.e., window size) for making those predictions remain to be determined.

## EXPERIMENTAL CHARACTERIZATION OF RBP-RNA INTERACTIONS

**Figure 2:**
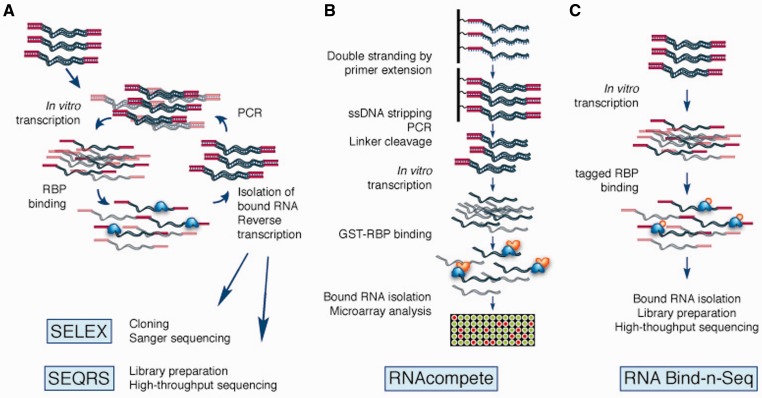
In *vitro* methods for determining RBP targets. (**A**) SELEX consists of several rounds of binding and amplification of RNA molecules. SEQRS modifies traditional SELEX by sequencing the bound pool of RNA at each round. (**B**) RNAcompete queries a designed RNA pool under competitive conditions and assays the bound RNAs using a microarray. (**C**) RNA Bind-n-Seq assays RNA binding by incubating RNA and various amounts of protein and sequencing the bound RNAs.

**Figure 3:**
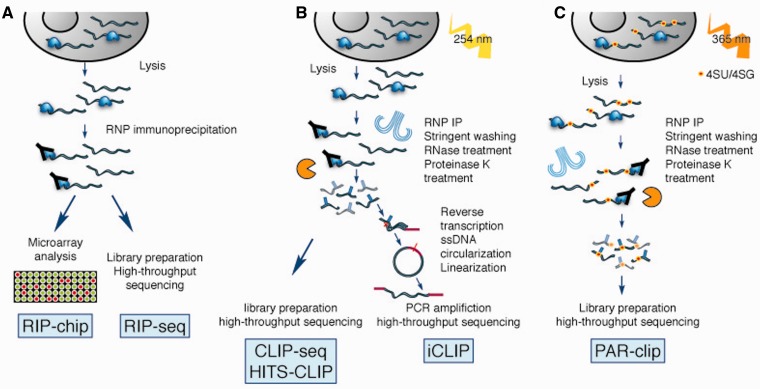
*In vivo* methods for determining RBP targets. (**A**) RIP-chip and RIP-seq determine bound RNAs by analyzing immunoprecipitated RNPs by microarrays or high-throughput sequencing. (**B**) UV cross-linking and immunoprecipitation allows more stringent washing and RNase treatment of bound RNAs. iCLIP identifies binding sites more precisely by taking advantage of the fact that the amino acid tag left by proteinase K treatment terminates reverse transcription. (**C**) PAR-CLIP is another modification of CLIP-seq that first treats the cell with a modified nucleoside (4SU or 6SG), which is incorporated into transcribed RNA. The modified nucleotide can be cross-linked using longer wavelength UV radiation.

High-throughput characterization of RBP–RNA interactions can be broken down into *in vitro* approaches (Figure 2), which determine the specificity of RBPs free from interacting proteins and other cellular factors, and *in vivo* approaches (Figure 3), which measure a snapshot of RBP binding to expressed RNAs. Here we also discuss some aspects of analysis of *in vivo* RBP-RNA data, as identifying bona fide protein binding sites can be challenging. Proteome-wide methods of identifying RBP–RNA interactions are described, and computational aspects of RNA motif finding are discussed.

### *In vitro* approaches

Systematic evolution of ligands by exponential enrichment (SELEX), also known as *in vitro* selection, is a common method for determining the consensus binding motif for an RBP [62, 63]. In a SELEX experiment, a pool of randomized RNA oligos is incubated with the RBP of interest, bound RNA is reverse transcribed, amplified by PCR and transcribed *in vitro*, and the process is repeated three or more times, with each selection increasing the proportion of high-affinity binding sites in the pool. Selected sequences are traditionally cloned and sequenced by Sanger sequencing. Although not strictly a high-throughput technique, SELEX has been used to ascertain high-affinity motifs for >70 metazoan proteins by various laboratories [14]. In addition, SELEX has been performed in parallel on a number of yeast RBPs [64]. While SELEX is a powerful technique for determining the optimal motif, the highest-affinity motif may not reflect the entirety of biologically functional binding sites [62, 63], and SELEX does not give quantitative information about the protein’s affinity for sub-optimal motifs.

SEQRS is a method that applies high-throughput sequencing to SELEX to monitor the enrichment of optimal and alternate binding motifs by sequencing after each round of selection [65]. Similar methods have been applied to measure the DNA-binding specificities of transcription factors in large numbers [66]. Using SEQRS, Campbell and colleagues determined the binding specificity of the *Caenorhabditis elegans* PUM domain RBP FBF-2 to a 20 nt randomized pool alone and in the presence of a non–RNA-binding peptide fragment of the CPEB protein CPB-1, and found that FBF-2’s binding specificity was altered in the presence of the CPB-1 fragment. As well, they reported an alternate binding mode for FBF-2 with a slightly different motif.

RNAcompete [67] is a method for determining the binding specificity of RBPs by incubating a purified GST-tagged RBP of interest with a pool of ∼40 nt RNAs designed to compactly and robustly represent all short sequences up to 9 bases. The binding reaction is performed with a vast excess of RNA so that RNA molecules compete for binding to the protein, and thus relative abundance can be used to assess relative affinity. After a single-step selection, the bound RNAs are washed, eluted and hybridized to a microarray for detection. RNAcompete was applied to a panel of 200 eukaryotic RBPs to determine their RNA sequence specificities [68]. Furthermore, protein sequence homology-based rules for predicting motifs of closely related RBPs were developed, allowing motifs for 30% of metazoan multi-RBD proteins to be inferred.

Neither SEQRS nor RNAcompete, in their current versions, are optimal for determining the structural specificity of RBPs. SEQRS uses a 20 nt pool, which is not long enough to encompass all but the simplest of secondary structures, while RNAcompete, owing to its microarray-based strategy, is able to robustly represent only primary RNA sequence in the ∼244 k RNA sequences in the pool, and so the current version of the RNAcompete pool was designed to avoid highly structured sequences. Nonetheless, motifs determined for proteins known to prefer hairpin loops, such as yeast Vts1p and human SNRPA and LIN28A, were determined, and correspond to the single-stranded portions (e.g. the loop portion of a hairpin loop) of the structural motif. Significant preferences of many RBPs for ssRNA and, in a few cases, hairpin loops were identified [68]. An alternative approach may be provided by the RNA Bind-n-Seq, in which a library of 40 nt RNAs is incubated with varying concentrations of protein, and bound RNAs are isolated and sequenced [69]. The greater representation of secondary structures in this starting pool may support more sensitive inference of the impact of RNA secondary structure on binding.

To study the kinetics of RBP binding to RNA sequences in a high-throughput manner, a recent study from the Greenleaf laboratory adapted an Illumina sequencing machine to measure binding affinity of the MS2 coat protein to ∼120 000 RNA sequences [70] representing a range of mutations to the consensus hairpin motif. Direct measurements of off-rates to >3000 sequences were observed, and the detailed data also enabled decomposition of the sequence and structural determinants of binding affinity at each base pair of the hairpin structure.

### *In vivo* approaches

A series of developments over the past decade have revolutionized determination of the RNAs bound *in vivo* by an RBP. The first genome-wide analyses were microarray based, and involved immunoprecipitation of RBP–RNA complexes using an antibody to the endogenous protein or to an epitope tag (denoted as RNA immunoprecipitation followed by microarray analysis, RIP-chip or high-throughput sequencing, RIP-seq). Tenenbaum et al were the first to apply RIP-chip to the determination of the RNA fraction bound to HuB, HuA/HuR, eIF-4 E and PABP [71]. Since then, RIP-chip/seq has been applied to determine the bound RNA complement of dozens of proteins in several species (collected in RBPDB [14] and reviewed in [72]), leading to the observation that RBP-to-mRNA interactions are, in general, many-to-many: each RBP interacts with a number of mRNAs, and each mRNA is regulated by at least several RBPs [73].

Although RIP allows identification of the target RNA molecules binding to an RBP, the data may include indirectly bound sequences, and precise locations of the binding site on the target mRNA may be difficult to determine. Additionally, RIP conditions must be calibrated to minimize reassociation of RBPs with mRNA *in vitro* after cell lysis, which has been observed under some conditions [74] but not others [75]. Cross-linking the RBP to the RNA using UV radiation before immunoprecipitation (CLIP) provides a way of both ensuring that *in vivo* contacts are maintained, as well as narrowing down the binding site [76]. UV cross-linking creates covalent bonds between proteins and RNA within a range of a few Ångstroms [77], and so background can be reduced by stringent purification protocols. Coupling of CLIP to high-throughput sequencing (HITS-CLIP or CLIP-seq) discloses RBP binding sites genome-wide. While resolution of CLIP-seq is generally limited to ∼30–60 nt [78] based on the length of the cross-linked RNA molecules after fragmentation, digestion of the cross-linked protein leaves an amino acid ‘tag’ on the RNA sequence, which can occasionally cause the reverse transcriptase to skip or misread the cross-linked base, producing mutations that can be diagnostic of the binding site [78]. The behavior of reverse transcriptase at the cross-linked nucleotide is also exploited by iCLIP, which takes advantage of the fact that often the amino acid tag causes termination of reverse transcription at that site to determine the precise location of the termination (and thus the cross-linking) site [79]. Precise determinations of binding site locations are also enabled by PAR-CLIP [31]. In PAR-CLIP, cells are exposed to a modified nucleoside such as 4-thiouridine (4SU) or 6-thioguanosine (6SG), which cross-links more efficiently with proteins at 365 nm UV light (as compared with the 254 nm UV light used for basic CLIP). The reverse transcriptase misreads the modified uridine, causing T → C conversions in the sequenced reads that can be used to pinpoint binding sites.

CLIP-seq and its variants are not without biases. UV cross-linking preferentially bonds certain nucleotides and certain amino acids [80], and not all proteins will cross-link effectively, possibly because of the absence of aromatic amino acids close to the RNA binding site [35, 81]. A recent study quantified UV cross-linking sensitivity by calculating the ratio of RNase-sensitive radioactive signal to protein abundance for a number of yeast RBPs, and observed a wide range of cross-linking efficiencies, even for RRM domains [82]. The 365 nm UV light used in PAR-CLIP only creates bonds at the modified base, so PAR-CLIP tags will tend to be enriched at locations with several of that base. This can bias motif finding toward U-rich motifs (in the context of 4SU PAR-CLIP) [83], as opposed to motifs derived from *in vitro* affinity measurements [84]. UV cross-linking has been applied to a number of systems including suspensions of mouse brain cells [2], and whole *C. elegans *[85] animals, whereas PAR-CLIP is usually applied to cells in culture that can efficiently take up the modified nucleosides, although it has also been performed in the *C. elegans* germ line [86].

Recently, Friedersdorf and Keene demonstrated that a large fraction (up to 45%) of reads (including high-abundance sites) from published PAR-CLIP data sets overlap with binding regions observed in background data from FLAG-GFP immunoprecipitations [87]. Background sites included T → C conversions, albeit at a lower rate, and similar background profiles were observed in several published data sets, suggesting that the background is a result of cross-linking proteins other than the target RBP. PAR-CLIP data from novel RBPs or small data sets had a higher fraction of background overlap. Although motifs determined *in vitro* are enriched in CLIP-seq reads [68], often motifs are not extractable from the CLIP-seq data *de novo *[88]. Background subtraction as described by Friedersdorf and Keene could enrich for the presence of known motifs and improve motif finding [87].

#### CLIP/PAR-CLIP data analysis

Identification of protein-bound sites from CLIP-seq (and variants) data is nontrivial. Because transcript abundance varies, ascertainment of the protein’s preference for a target RNA through quantitation of the number of CLIP-seq reads mapping to a transcript sequence requires normalization to transcript abundance. Additionally, the choice of RNase and conditions chosen to fragment the cross-linked protein–RNA complex has significant impact on the base composition of the observed CLIP tags [83]. To overcome these challenges, several strategies have been used. CIMS [78] uses the diagnostic deletions present in CLIP-seq tags to help pinpoint the location of RBP binding site by clustering reads and identifying reproducibly occurring deletions. PARalyzer [89] uses the number of T → C conversions that are diagnostic of PAR-CLIP binding sites to define the limits of the binding site using a kernel density-based classifier, but does not use transcript abundance data and so generates a set of RBP-bound sites rather than RBP target preferences. Uren and colleagues [90] directly modeled the background distribution of read counts and account for mappability and transcript abundance (and, optionally, data from a contrasting experiment for comparison of differential binding). These methods, the production of additional data sets for comparison, and the incorporation of control background data [87] may allow for more precise quantitation of binding activity from CLIP data, which could reflect the stability or half-life of RBP-RNA interactions, and provide quantitative data to aid motif finding.

### Proteome-wide approaches

While the majority of recent efforts have focused on identification of the RNAs bound to a given RBP, mass spectrometry has allowed complementary approaches to discover new RBPs and determine all the RBPs binding an RNA. In addition, modification of the *in vivo* methods discussed above to be protein-agnostic allows the identification of all the protein-bound sites in expressed transcripts.

#### Identification of RBPs proteome wide

Proteomics approaches have been applied to identify a more complete complement of RBPs in eukaryotic genomes, which is important because there are a number of RBPs without canonical RNA-binding domains [91, 92]. In yeast, probing protein microarrays with labeled RNA revealed a number of putative novel RBPs, many which already bear annotations as enzymes [93, 94]. More recently, two studies cross-linked proteins to RNA and applied an oligo (dT) pulldown and mass spectrometry to identify proteins contained in ribonucleoprotein complexes in human cells [35, 95]. Both studies identified ∼800 RNA-associated proteins, several of which they validated by PAR-CLIP [95] and a fluorescence-based *in vivo* binding assay [35]. Surprisingly ∼250–300 of these 800 do not contain classical RBDs or bear previous functional annotation as RBPs. A wide variety of proteins were identified in these studies, including several enzymes involved in intermediary metabolism. However, in contrast to the previous studies in yeast, metabolic enzymes were overall depleted in the set of RNA-associated proteins identified in both studies. Despite this, further *in vitro* or *in vivo* analyses are required to establish whether or not these novel RBPs simply bind RNA non-specifically as the PAR-CLIP data from many of the RBPs resembles background data derived from FLAG-GFP immunoprecipitations [87].

#### Application of mass spectrometry to determine proteins targeting a specific RNA

Complementary to the RBP-centric approaches described above, development of mass spectrometric methods for determining the protein complement of diverse mixtures has allowed the direct ascertainment of proteins bound to an RNA sequence. Most approaches have been performed *in vitro*, and involve tethering the target RNA molecule to a solid support by chemical modification of the RNA [96] or using an RNA aptamer to a protein that can then be either immunoprecipitated or attached to a solid support such as streptavidin [97] and incubating it with cellular lysate. Purification of *in vivo* assembled RNPs has been accomplished using oligonucleotides complementary to the RNA sequence [98], coexpression of MS2 coat protein and the RNA sequence harbouring an MS2 aptamer [99], and delivery of a peptide nucleic acid (PNA) complementary to the target RNA sequence and bonded to a photoactivatable reagent that will form cross-links between the PNA and nearby proteins [100]. Although quantitative mass spectrometry-based methods are less well developed than high-throughput sequencing approaches, these techniques have been used to identify RBP regulators of a telomere-associated noncoding RNA [101] and to classify a conserved RNA secondary structure predicted using a covariation approach as a putative internal ribosome entry site [97], demonstrating their value as assays to probe the functional significance of predicted RNA motifs. Interestingly, a SILAC-based mass spectrometry approach has been used to investigate binding sites determined using PAR-CLIP and confirmed many of the binding sites, a direct demonstration of the complementarity of the approaches [102].

#### Protein occupancy profiling

Several methods have been developed for discovering protein-bound sites on RNAs agnostic of the RBP, denoted protein occupancy (or interaction) profiling. These methods function similarly to PAR-CLIP, except that instead of immunoprecipitation of a target RBP, RNPs are purified using oligo (dT) beads [95] or chemical biotinylation of proteins [103]. High-throughput sequencing identifies bound sites. Silverman and colleagues applied a slightly different approach: cross-linking with formaldehyde, digesting RNA using RNases, reversing the cross-links and sequencing the resulting RNA [104]. This method uncovered fewer binding sites overall but was not limited to processed mRNAs and so observed sites on introns and non-polyadenylated RNAs. The method of Baltz and colleagues has also been applied to MCF7 cells [105], allowing observation of overall differences in binding site occupancy by RBPs between cell types. Interestingly, binding sites with increased occupancy in MCF7 cells contained predicted binding sites for the ELAV family of ARE-binding proteins, regulators of mRNA stability that have been previously implicated with carcinogenesis and poor prognosis [106, 107], and mRNAs with differentially occupied sites had longer half-lives in MCF7 cells, suggesting a widespread role for this protein family in post-transcriptional regulation in cancer cells [105].

### Computational methods for examining protein-RNA interactions

The goal of complete understanding of the functions of RBPs in post-transcriptional regulation requires computational analysis of RBP-RNA interactions to interpret experimental data and model how RBPs find and bind to their targets. A general strategy for predicting RBP binding involves (1) motif finding and (2) prediction of binding sites using the discovered motifs. Online databases collecting RBP motifs and *in vivo* data will also be described.

#### Motif finding

Learning RBP binding motifs can be accomplished by applying DNA motif finders, which only consider the RNA sequence, or by considering RNA secondary structure either explicitly or as a layer on top of sequence preference. The basic approaches are summarized in Table 1. The DNA-based methods MEME, PhyloGibbs and cERMIT have been used to identify motifs from RIP-chip (both raw, and filtered to include only sequences with enriched hexamers), CLIP-seq and PAR-CLIP data [64, 84, 108–110]. Despite ignoring secondary structure, these methods are often successful, presumably because many RBPs fundamentally bind short ssRNA sequences (5–10 nt), often without variable gaps between bound segments that can confound standard position weight matrix (PWM) based methods.

**Table 1:** Motif-finding algorithms used for analyzing RBP-RNA interaction data

Algorithm	Input	Type of motif generated	Considers secondary structure?	Reference
MEME	Positive (and optionally, negative) sequences	PWM	No	[111]
PhyloGibbs	Positive (and optionally, negative) sequences	PWM	No	[112]
REFINE	Positive sequences	N/A, Filtering procedure to only consider sequences containing three enriched hexamers; filtered sequences are then submitted to another motif finding algorithm	No	[64]
cERMIT	Rank ordered sequences	PWM	No	[113]
DRIMUST	Rank ordered sequences	IUPAC motif, possibly gapped	No	[114]
StructuRED	Positive and negative sequences	PWM in a hairpin loop	Yes, considers possible hairpin loops up to 7 bases with at least 3 paired bases	[115]
TEISER	Sequences and scores (e.g., stability scores)	PWM in a hairpin loop	Yes, considers possible hairpin loops with stems 4-7 bases long and loop sizes of 4-9 bases	[116]
RNAcontext	Sequences and affinity scores	PWM with structural context scores	Yes, learns the preferred structural context of each base in a motif	[117]
GraphProt	Positive and negative sequences	graph-based sequence and structure motifs, can be visualized with logos	Yes, models RNA structure using a graph-based encoding	[118]
CMfinder	Positive sequences	structured sequence	Yes, SCFG-based, examines the most stable structures in the input	[119]
RNApromo	Positive sequences	structured sequence	Yes, SCFG-based, optimizes a motif from an initial set of substructures generated from the input	[120]
#ATS	Positive and negative sequences	IUPAC	Yes, scores candidate binding sites by accessibility	[121]
MEMERIS	Positive and negative sequences	PWM	Yes, uses accessibility as prior knowledge to guide motif finding toward single-stranded regions	[122]

Specialized methods that incorporate RNA secondary structure generally break down into two camps: the first is based on determining the linear structural context around a sequence motif. MEMERIS is an extension of the popular MEME algorithm that uses RNA accessibility as a prior probability to guide motif finding to single-stranded regions [122]. Similarly, Li and colleagues applied accessibility to select motifs that best distinguish bound and unbound transcripts [121]. MatrixREDUCE input is filtered to include only possible hairpin loop structures as part of StructRED [115]. Finally, RNAcontext models the probability that each base in a motif is in a particular secondary structure context (e.g. a hairpin loop) and learns the weights for each position from a set of input sequences annotated with relative binding affinity [117]. RNAcontext is also available on the RBPmotif Web server [123].

The second approach considers RNA secondary structure explicitly and includes stochastic context-free grammar (SCFG) and graphical approaches. CMfinder [119] and RNApromo [120] both start with a set of structures predicted using thermodynamic methods. RNApromo was used to predict motifs in sets of RNAs bound by RBPs in yeast [120]. Maticzka and colleagues developed the graph kernel-based GraphProt, and applied it to learn motifs from CLIP-seq data [118], producing motifs that were highly predictive of binding: the certainty of predicted motifs for PTB correlated with measured RBP affinity.

The fact that many RBPs have multiple RNA binding domains raises the interesting possibility of RBPs binding to bipartite or complex motifs. Gapped motifs have been described for PTB (4 RRM domains, [124]) and the STAR family of RBPs (1 KH domain, but the proteins bind as a dimer, [125]). Motif finding algorithms that incorporate gapped positions have been developed [126] but have not been extensively applied to RNA–protein interaction data. Exceptions include Leibovich and Yakhini, who applied the DRIMUST algorithm to a RIP-chip data set of yeast PUM-HD proteins, several of which displayed gapped motifs [114], and hidden Markov model-based approaches for detecting clustered binding sites in PTB [127] and Nova/Mbnl [128] data.

#### Databases

Online databases that collect RNA–protein interactions are summarized in Table 2. The RBP DataBase (RBPDB) stores low- and high-throughput experimental evidence of RNA-binding for metazoan RBPs [14]. The Catalogue of Inferred Sequence Binding Preference of RNA binding proteins (CISBP-RNA) focuses on sequence motifs, and includes inference of motifs for RBPs homologous to a studied protein [68]. *In vivo* RBP binding sites are catalogued in three databases, starBase [129], doRiNA [130], and CLIPz [131]. These databases integrate high-throughput CLIP, PAR-CLIP, and iCLIP data with other data such as miRNA binding sites, and offer tools for online analysis.

**Table 2:** Databases that collect RNA-protein interactions

Database	URL	Features	Reference
RBPDB	http://rbpdb.ccbr.utoronto.ca/	Direct observations of protein-RNA interactions in metazoans, both low- and high-throughput	[14]
CISBP-RNA	http://cisbp-rna.ccbr.utoronto.ca/	Directly observed and predicted (by homology with known proteins) motifs. Tools for scanning sequences and comparing motifs	[68]
starBase	http://starbase.sysu.edu.cn/	RBP-RNA and miRNA-RNA interactions from CLIP data	[129]
doRiNa	http://dorina.mdc-berlin.de/	mRNA-centric or RBP-centric search of CLIP data including combinatorial search	[130]
CLIPz	http://www.clipz.unibas.ch/	Storage and analysis (mapping reads, extracting clusters, mapping T→C conversions) of CLIP data	[131]

## OUTLOOK

High-throughput identification of protein–RNA interactions has improved understanding of the targets of RBPs in diverse cellular contexts, and focus is shifting from cataloging RNA–protein interactions to understanding the individual and combined effects of RBPs on global RNA metabolism and gene expression. Methods to produce extensive functional data sets of RNA splicing [132], stability [133] and translation [134] have been developed, and more data are likely on the way. High-throughput methods to assay the effect of sequence features in 3′ and 5′ UTRs on RNA expression levels will help generate training datasets to learn subtle features of complex or combinatorial regulation [135–137]. Computational analysis and modeling will undoubtedly play a primary role in understanding RNA biology in the near future.

**Key points**

- RBPs apply different strategies for binding RNA.
- For certain RBPs, RNA secondary structure is a key component of RNA binding, but widespread prediction and measurement of RNA secondary structure remains difficult especially due to the potential impact of other RBPs on mRNA structure.
- *In vitro* binding specificity of RBPs can be determined using microarray-based methods (such as RNAcompete) or high-throughput sequencing-based methods (such as RNA Bind-n-Seq).
- *In vivo* targets of RBPs are determined using CLIP-seq/HITS-clip, and precise binding sites are more easily defined using the PAR-CLIP and iCLIP variants.
- A general strategy for predicting RBP binding involves (1) motif finding and (2) prediction of binding sites using the discovered motifs.

## FUNDING

K.B.C. is supported by an NSERC Alexander Graham Bell Canada Graduate Scholarship. This work was funded by Canadian Institute for Health Research operating grant to Q.D.M. and T.R.H. MOP-125894.
